# Supplementary material for: Clinicopathological value of long non-coding RNA profiles in gastrointestinal stromal tumor
Source: PeerJ. 2021 Sep 3;9:e11946. doi: 10.7717/peerj.11946 (PMC8420874; doi:10.7717/peerj.11946)
Supplement: Supplemental Information 1 [file peerj-09-11946-s001.docx]

Supplementary table 1 Case summary from microarray data GSE8167 and GSE17743

|  | | | Age | diameter | Gender | Location | mutation | Sample_ geo_accession |
| --- | --- | --- | --- | --- | --- | --- | --- | --- |
| LncRNAprobeid_ GIST61_k4 | G1 | 1 | 61 | 3 | female | intest | KIT | GSM202197 |
|  |  | 2 | 64 | 4 | female | stomac | WT | GSM202198 |
|  |  | 3 | 64 | 4 | female | stomac | WT | GSM202199 |
|  |  | 4 | 40 | 10 | female | stomac | KIT | GSM202205 |
|  |  | 5 | 55 | 6 | female | stomac | WT | GSM202207 |
|  |  | 6 | 69 | 20 | female | stomac | KIT | GSM202208 |
|  |  | 7 | 63 | 13 | female | stomac | KIT | GSM202212 |
|  |  | 8 | 41 | 4 | female | stomac | KIT | GSM202217 |
|  |  | 9 | 52 | 5 | female | stomac | WT | GSM202218 |
|  |  | 10 | 72 | 25 | female | stomac | KIT | GSM202220 |
|  |  | 11 | 68 | 6 | female | intest | WT | GSM202227 |
|  |  | Total (n) | 11 | 11 | 11 | 11 | 11 | 11 |
|  | G2 | 1 | 68 | 19 | male | stomac | KIT | GSM202200 |
|  |  | 2 | 54 | 10 | male | stomac | KIT | GSM202201 |
|  |  | 3 | 68 | 4 | male | intest | KIT | GSM202202 |
|  |  | 4 | 77 | 4 | male | intest | WT | GSM202203 |
|  |  | 5 | 56 | 38 | male | stomac | KIT | GSM202204 |
|  |  | 6 | 58 | 13 | male | stomac | KIT | GSM202206 |
|  |  | 7 | 50 | 21 | male | stomac | WT | GSM202209 |
|  |  | 8 | 51 | 12 | male | stomac | KIT | GSM202210 |
|  |  | 9 | 71 | 6 | male | stomac | KIT | GSM202211 |
|  |  | 10 | 52 | 7 | male | stomac | KIT | GSM202213 |
|  |  | 11 | 76 | 7 | male | intest | KIT | GSM202214 |
|  |  | 12 | 34 | 18 | male | intest | KIT | GSM202215 |
|  |  | 13 | 81 | 6 | male | stomac | KIT | GSM202216 |
|  |  | 14 | 68 | 7 | male | intest | KIT | GSM202219 |
|  |  | 15 | 68 | 17 | male | stomac | KIT | GSM202221 |
|  |  | 16 | 82 | 4 | male | intest | KIT | GSM202222 |
|  |  | 17 | 61 | 8 | male | stomac | WT | GSM202223 |
|  |  | 18 | 61 | 12 | female | intest | KIT | GSM202224 |
|  |  | 19 | 69 | 7 | male | stomac | WT | GSM202225 |
|  |  | 20 | 47 | 4 | male | stomac | KIT | GSM202226 |
|  |  | Total (n) | 20 | 20 | 20 | 20 | 20 | 20 |
|  | G3 | 1 | 71 | 11 | male | stomac | KIT | GSM202228 |
|  |  | 2 | . | 40 | Male | stomac | KIT | GSM442936 |
|  |  | 3 | . | 35 | Female | stomac | KIT | GSM442938 |
|  |  | 4 | . | 30 | Male | stomac | KIT | GSM442941 |
|  |  | 5 | . | 35 | Female | stomac | WT | GSM442944 |
|  |  | 6 | . | 70 | Male | stomac | KIT | GSM442945 |
|  |  | 7 | . | 95 | Male | stomac | PDGFRA | GSM442948 |
|  |  | 8 | . | 35 | Male | stomac | PDGFRA | GSM442949 |
|  |  | 9 | . | 65 | Male | stomac | PDGFRA | GSM442955 |
|  |  | 10 | . | 80 | Male | stomac | PDGFRA | GSM442961 |
|  |  | 11 | . | 40 | Female | stomac | KIT | GSM442964 |
|  |  | Total (n) | 1 | 11 | 11 | 11 | 11 | 11 |
|  | G4 | 1 | . | 53 | Male | stomac | KIT | GSM442937 |
|  |  | 2 | . | 35 | Male | stomac | PDGFRA | GSM442939 |
|  |  | 3 | . | 18 | Male | stomac | KIT | GSM442940 |
|  |  | 4 | . | 55 | Male | stomac | KIT | GSM442942 |
|  |  | 5 | . | 55 | Male | stomac | WT | GSM442943 |
|  |  | 6 | . | 20 | Female | stomac | KIT | GSM442946 |
|  |  | 7 | . | 70 | Male | stomac | PDGFRA | GSM442947 |
|  |  | 8 | . | 60 | Male | stomac | PDGFRA | GSM442950 |
|  |  | 9 | . | 90 | Male | stomac | WT | GSM442951 |
|  |  | 10 | . | 50 | Male | stomac | PDGFRA | GSM442952 |
|  |  | 11 | . | 25 | Female | stomac | PDGFRA | GSM442953 |
|  |  | 12 | . | 160 | Male | stomac | KIT | GSM442954 |
|  |  | 13 | . | 40 | Male | stomac | PDGFRA | GSM442956 |
|  |  | 14 | . | 65 | Male | stomac | KIT | GSM442957 |
|  |  | 15 | . | 15 | Male | stomac | KIT | GSM442958 |
|  |  | 16 | . | 70 | Male | stomac | KIT | GSM442959 |
|  |  | 17 | . | 28 | Male | stomac | KIT | GSM442960 |
|  |  | 18 | . | 30 | Female | stomac | KIT | GSM442962 |
|  |  | 19 | . | 90 | Female | stomac | PDGFRA | GSM442963 |
|  |  | Total (n) |  | 19 | 19 | 19 | 19 | 19 |
|  | Total (n) |  | 32 | 61 | 61 | 61 | 61 | 61 |
