# Supplementary material for: Clinicopathological value of long non-coding RNA profiles in gastrointestinal stromal tumor
Source: PeerJ. 2021 Sep 3;9:e11946. doi: 10.7717/peerj.11946 (PMC8420874; doi:10.7717/peerj.11946)
Supplement: Supplemental Information 2 — The raw data source of gastrointestinal stromal tumors patients analysed in this article. [file peerj-09-11946-s002.docx]

GIST & [Homo sapiens](https://www.ncbi.nlm.nih.gov/Taxonomy/Browser/wwwtax.cgi?mode=Info&id=9606) & [HG-U133_Plus_2] Affymetrix Human Genome U133 Plus 2.0 Array,

**29samples GSE17743** Public on Aug 21, 2009

**32samples GSE8167** Public on Jun 15, 2008

**25samples GSE20709** Public on Aug 27, 2010

**29samples GSE17743**

<https://www.ncbi.nlm.nih.gov/geo/query/acc.cgi?acc=GSE17743>

| Status | Public on Aug 21, 2009 |
| --- | --- |
| Title | Gene expression profiles differentiating gastrointestinal stromal tumours according to KIT mutations and expression |
| Organism | [Homo sapiens](https://www.ncbi.nlm.nih.gov/Taxonomy/Browser/wwwtax.cgi?mode=Info&id=9606) |
| Experiment type | Expression profiling by array |
| Summary | Gastrointestinal stromal tumours (GISTs) represent a heterogeneous group of tumours of mesenchymal origin characterized by gain-of-function mutations in KIT or PDGFRA of the type III receptor tyrosine kinase family. Although mutations in either receptor are thought to drive an early oncogenic event through similar pathways, two previous studies reported the mutation-specific gene expression profiles. However, their further conclusions were rather discordant. To clarify the molecular characteristics of differentially expressed genes according to GIST receptor mutations, we combined microarray-based analysis with detailed functional annotations. |
|  |  |
| Overall design | 29 samples: 15 with KIT mutation detected, 11 with PDGFRA mutation detected, 3 with no mutation detected |
|  |  |
| Contributor(s) | [Ostrowski J](https://www.ncbi.nlm.nih.gov/pubmed/?term=Ostrowski%20J%5bAuthor%5d), [Polkowski M](https://www.ncbi.nlm.nih.gov/pubmed/?term=Polkowski%20M%5bAuthor%5d), [Paziewska A](https://www.ncbi.nlm.nih.gov/pubmed/?term=Paziewska%20A%5bAuthor%5d), [Skrzypczak M](https://www.ncbi.nlm.nih.gov/pubmed/?term=Skrzypczak%20M%5bAuthor%5d), [Goryca K](https://www.ncbi.nlm.nih.gov/pubmed/?term=Goryca%20K%5bAuthor%5d), [Rubel T](https://www.ncbi.nlm.nih.gov/pubmed/?term=Rubel%20T%5bAuthor%5d), [Kokoszyńska K](https://www.ncbi.nlm.nih.gov/pubmed/?term=Kokoszyńska%20K%5bAuthor%5d), [Rutkowski P](https://www.ncbi.nlm.nih.gov/pubmed/?term=Rutkowski%20P%5bAuthor%5d), [Nowecki ZI](https://www.ncbi.nlm.nih.gov/pubmed/?term=Nowecki%20ZI%5bAuthor%5d), [Jerzak A](https://www.ncbi.nlm.nih.gov/pubmed/?term=Jerzak%20A%5bAuthor%5d), [Jarosz D](https://www.ncbi.nlm.nih.gov/pubmed/?term=Jarosz%20D%5bAuthor%5d), [Ruka W](https://www.ncbi.nlm.nih.gov/pubmed/?term=Ruka%20W%5bAuthor%5d), [Wyrwicz LS](https://www.ncbi.nlm.nih.gov/pubmed/?term=Wyrwicz%20LS%5bAuthor%5d) |
| Citation(s) | - Ostrowski J, Polkowski M, Paziewska A, Skrzypczak M et al. Functional features of gene expression profiles differentiating gastrointestinal stromal tumours according to KIT mutations and expression. BMC Cancer 2009 Nov 27;9:413. PMID: [19943934](https://www.ncbi.nlm.nih.gov/pubmed/19943934) |
| Submission date | Aug 20, 2009 |
| Last update date | Mar 25, 2019 |
| Contact name | Krzysztof Goryca |
| Organization name | Centrum Onkologii Instytut im. Marii Skłodowskiej Curie |
| Department | Department of Genetics |
| Street address | Roentgena 5 |
| City | Warszawa |
| ZIP/Postal code | 02-781 |
| Country | Poland |
|  |  |
| Platforms (1) | \| [GPL570](https://www.ncbi.nlm.nih.gov/geo/query/acc.cgi?acc=GPL570) \| [HG-U133_Plus_2] Affymetrix Human Genome U133 Plus 2.0 Array \| \| --- \| --- \| |
| Samples (29)  [[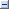](javascript:HandleVisibilityChangeL472411727()) Less...](javascript:HandleVisibilityChangeL472411727())  [[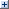](javascript:HandleVisibilityChangeL472411727()) More...](javascript:HandleVisibilityChangeL472411727()) | \| [GSM442936](https://www.ncbi.nlm.nih.gov/geo/query/acc.cgi?acc=GSM442936) \| GIST_KIT_1 \| \| --- \| --- \| \| [GSM442937](https://www.ncbi.nlm.nih.gov/geo/query/acc.cgi?acc=GSM442937) \| GIST_KIT_2 \| \| [GSM442938](https://www.ncbi.nlm.nih.gov/geo/query/acc.cgi?acc=GSM442938) \| GIST_KIT_3 \|  \| [GSM442939](https://www.ncbi.nlm.nih.gov/geo/query/acc.cgi?acc=GSM442939) \| GIST_PDGFRA_1 \| \| --- \| --- \| \| [GSM442940](https://www.ncbi.nlm.nih.gov/geo/query/acc.cgi?acc=GSM442940) \| GIST_KIT_4 \| \| [GSM442941](https://www.ncbi.nlm.nih.gov/geo/query/acc.cgi?acc=GSM442941) \| GIST_KIT_5 \| \| [GSM442942](https://www.ncbi.nlm.nih.gov/geo/query/acc.cgi?acc=GSM442942) \| GIST_KIT_6 \| \| [GSM442943](https://www.ncbi.nlm.nih.gov/geo/query/acc.cgi?acc=GSM442943) \| GIST_WT_1 \| \| [GSM442944](https://www.ncbi.nlm.nih.gov/geo/query/acc.cgi?acc=GSM442944) \| GIST_WT_2 \| \| [GSM442945](https://www.ncbi.nlm.nih.gov/geo/query/acc.cgi?acc=GSM442945) \| GIST_KIT_7 \| \| [GSM442946](https://www.ncbi.nlm.nih.gov/geo/query/acc.cgi?acc=GSM442946) \| GIST_KIT_8 \| \| [GSM442947](https://www.ncbi.nlm.nih.gov/geo/query/acc.cgi?acc=GSM442947) \| GIST_PDGFRA_2 \| \| [GSM442948](https://www.ncbi.nlm.nih.gov/geo/query/acc.cgi?acc=GSM442948) \| GIST_PDGFRA_3 \| \| [GSM442949](https://www.ncbi.nlm.nih.gov/geo/query/acc.cgi?acc=GSM442949) \| GIST_PDGFRA_4 \| \| [GSM442950](https://www.ncbi.nlm.nih.gov/geo/query/acc.cgi?acc=GSM442950) \| GIST_PDGFRA_5 \| \| [GSM442951](https://www.ncbi.nlm.nih.gov/geo/query/acc.cgi?acc=GSM442951) \| GIST_WT_3 \| \| [GSM442952](https://www.ncbi.nlm.nih.gov/geo/query/acc.cgi?acc=GSM442952) \| GIST_PDGFRA_6 \| \| [GSM442953](https://www.ncbi.nlm.nih.gov/geo/query/acc.cgi?acc=GSM442953) \| GIST_PDGFRA_7 \| \| [GSM442954](https://www.ncbi.nlm.nih.gov/geo/query/acc.cgi?acc=GSM442954) \| GIST_KIT_9 \| \| [GSM442955](https://www.ncbi.nlm.nih.gov/geo/query/acc.cgi?acc=GSM442955) \| GIST_PDGFRA_8 \| \| [GSM442956](https://www.ncbi.nlm.nih.gov/geo/query/acc.cgi?acc=GSM442956) \| GIST_PDGFRA_9 \| \| [GSM442957](https://www.ncbi.nlm.nih.gov/geo/query/acc.cgi?acc=GSM442957) \| GIST_KIT_10 \| \| [GSM442958](https://www.ncbi.nlm.nih.gov/geo/query/acc.cgi?acc=GSM442958) \| GIST_KIT_11 \| \| [GSM442959](https://www.ncbi.nlm.nih.gov/geo/query/acc.cgi?acc=GSM442959) \| GIST_KIT_12 \| \| [GSM442960](https://www.ncbi.nlm.nih.gov/geo/query/acc.cgi?acc=GSM442960) \| GIST_KIT_13 \| \| [GSM442961](https://www.ncbi.nlm.nih.gov/geo/query/acc.cgi?acc=GSM442961) \| GIST_PDGFRA_10 \| \| [GSM442962](https://www.ncbi.nlm.nih.gov/geo/query/acc.cgi?acc=GSM442962) \| GIST_KIT_14 \| \| [GSM442963](https://www.ncbi.nlm.nih.gov/geo/query/acc.cgi?acc=GSM442963) \| GIST_PDGFRA_11 \| \| [GSM442964](https://www.ncbi.nlm.nih.gov/geo/query/acc.cgi?acc=GSM442964) \| GIST_KIT_15 \| |
| **Relations** | |
| BioProject | [PRJNA118375](https://www.ncbi.nlm.nih.gov/bioproject/PRJNA118375) |

**32samples GSE8167**

<https://www.ncbi.nlm.nih.gov/geo/query/acc.cgi?acc=GSE8167>

| Status | Public on Jun 15, 2008 |
| --- | --- |
| Title | Distinct gene-expression-defined classes of gastrointestinal stromal tumor (GIST). |
| Organism | [Homo sapiens](https://www.ncbi.nlm.nih.gov/Taxonomy/Browser/wwwtax.cgi?mode=Info&id=9606) |
| Experiment type | Expression profiling by array |
| Summary | GIST is considered to invariably arise through gain-of-function KIT or PDGFRA mutation of the interstitial cells of Cajal (ICC). However, the genetic basis of the malignant progression of GIST is poorly understood. We analysed the expression levels of 54,613 probe sets in 32 surgical samples of untreated GIST of the stomach and small intestine with GeneChip Human Genome U133 Plus 2.0 arrays. Keywords: gene expression array-based, count |
|  |  |
| Overall design | Total RNA was extracted from 32 fresh frozen tumour specimens. We analysed the global gene exprssion profiles of these GIST cases in order to clarify the genomic basis behind the malignant progression of this tumor |
|  |  |
| Citation(s) | - Yamaguchi U, Nakayama R, Honda K, Ichikawa H et al. Distinct gene expression-defined classes of gastrointestinal stromal tumor. J Clin Oncol 2008 Sep 1;26(25):4100-8. PMID: [18757323](https://www.ncbi.nlm.nih.gov/pubmed/18757323) |
| Submission date | Jun 18, 2007 |
| Last update date | Mar 25, 2019 |
| Contact name | Umio Yamaguchi |
| E-mail(s) | [umioy@yahoo.co.jp](mailto:umioy@yahoo.co.jp) |
| Organization name | NCCRI |
| Department | Chemotherapy |
| Street address | 5-1-1, Tsukiji |
| City | Chuo |
| State/province | Tokyo |
| ZIP/Postal code | 104-0045 |
| Country | Japan |
|  |  |
| Platforms (1) | \| [GPL570](https://www.ncbi.nlm.nih.gov/geo/query/acc.cgi?acc=GPL570) \| [HG-U133_Plus_2] Affymetrix Human Genome U133 Plus 2.0 Array \| \| --- \| --- \| |
| Samples (32)  [[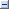](javascript:HandleVisibilityChangeL183099571()) Less...](javascript:HandleVisibilityChangeL183099571())  [[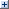](javascript:HandleVisibilityChangeL183099571()) More...](javascript:HandleVisibilityChangeL183099571()) | \| [GSM202197](https://www.ncbi.nlm.nih.gov/geo/query/acc.cgi?acc=GSM202197) \| Small intestinal GIST 1 \| \| --- \| --- \| \| [GSM202198](https://www.ncbi.nlm.nih.gov/geo/query/acc.cgi?acc=GSM202198) \| Gastric GIST 1 \| \| [GSM202199](https://www.ncbi.nlm.nih.gov/geo/query/acc.cgi?acc=GSM202199) \| Gastric GIST 2 \|  \| [GSM202200](https://www.ncbi.nlm.nih.gov/geo/query/acc.cgi?acc=GSM202200) \| Gastric GIST 3 \| \| --- \| --- \| \| [GSM202201](https://www.ncbi.nlm.nih.gov/geo/query/acc.cgi?acc=GSM202201) \| Gastric GIST 4 \| \| [GSM202202](https://www.ncbi.nlm.nih.gov/geo/query/acc.cgi?acc=GSM202202) \| Small intestinal GIST 2 \| \| [GSM202203](https://www.ncbi.nlm.nih.gov/geo/query/acc.cgi?acc=GSM202203) \| Small intestinal GIST 3 \| \| [GSM202204](https://www.ncbi.nlm.nih.gov/geo/query/acc.cgi?acc=GSM202204) \| Gastric GIST 5 \| \| [GSM202205](https://www.ncbi.nlm.nih.gov/geo/query/acc.cgi?acc=GSM202205) \| Gastric GIST 6 \| \| [GSM202206](https://www.ncbi.nlm.nih.gov/geo/query/acc.cgi?acc=GSM202206) \| Gastric GIST 7 \| \| [GSM202207](https://www.ncbi.nlm.nih.gov/geo/query/acc.cgi?acc=GSM202207) \| Gastric GIST 8 \| \| [GSM202208](https://www.ncbi.nlm.nih.gov/geo/query/acc.cgi?acc=GSM202208) \| Gastric GIST 9 \| \| [GSM202209](https://www.ncbi.nlm.nih.gov/geo/query/acc.cgi?acc=GSM202209) \| Gastric GIST 10 \| \| [GSM202210](https://www.ncbi.nlm.nih.gov/geo/query/acc.cgi?acc=GSM202210) \| Gastric GIST 11 \| \| [GSM202211](https://www.ncbi.nlm.nih.gov/geo/query/acc.cgi?acc=GSM202211) \| Gastric GIST 12 \| \| [GSM202212](https://www.ncbi.nlm.nih.gov/geo/query/acc.cgi?acc=GSM202212) \| Gastric GIST 13 \| \| [GSM202213](https://www.ncbi.nlm.nih.gov/geo/query/acc.cgi?acc=GSM202213) \| Gastric GIST 14 \| \| [GSM202214](https://www.ncbi.nlm.nih.gov/geo/query/acc.cgi?acc=GSM202214) \| Small intestinal GIST 4 \| \| [GSM202215](https://www.ncbi.nlm.nih.gov/geo/query/acc.cgi?acc=GSM202215) \| Small intestinal GIST 5 \| \| [GSM202216](https://www.ncbi.nlm.nih.gov/geo/query/acc.cgi?acc=GSM202216) \| Gastric GIST 15 \| \| [GSM202217](https://www.ncbi.nlm.nih.gov/geo/query/acc.cgi?acc=GSM202217) \| Gastric GIST 16 \| \| [GSM202218](https://www.ncbi.nlm.nih.gov/geo/query/acc.cgi?acc=GSM202218) \| Gastric GIST 17 \| \| [GSM202219](https://www.ncbi.nlm.nih.gov/geo/query/acc.cgi?acc=GSM202219) \| Small intestinal GIST 6 \| \| [GSM202220](https://www.ncbi.nlm.nih.gov/geo/query/acc.cgi?acc=GSM202220) \| Gastric GIST 18 \| \| [GSM202221](https://www.ncbi.nlm.nih.gov/geo/query/acc.cgi?acc=GSM202221) \| Gastric GIST 19 \| \| [GSM202222](https://www.ncbi.nlm.nih.gov/geo/query/acc.cgi?acc=GSM202222) \| Small intestinal GIST 7 \| \| [GSM202223](https://www.ncbi.nlm.nih.gov/geo/query/acc.cgi?acc=GSM202223) \| Gastric GIST 20 \| \| [GSM202224](https://www.ncbi.nlm.nih.gov/geo/query/acc.cgi?acc=GSM202224) \| Small intestinal GIST 8 \| \| [GSM202225](https://www.ncbi.nlm.nih.gov/geo/query/acc.cgi?acc=GSM202225) \| Gastric GIST 21 \| \| [GSM202226](https://www.ncbi.nlm.nih.gov/geo/query/acc.cgi?acc=GSM202226) \| Gastric GIST 22 \| \| [GSM202227](https://www.ncbi.nlm.nih.gov/geo/query/acc.cgi?acc=GSM202227) \| Small intestinal GIST 9 \| \| [GSM202228](https://www.ncbi.nlm.nih.gov/geo/query/acc.cgi?acc=GSM202228) \| Gastric GIST 23 \| |
| **Relations** | |
| BioProject | [PRJNA101063](https://www.ncbi.nlm.nih.gov/bioproject/PRJNA101063) |

<https://www.ncbi.nlm.nih.gov/geo/query/acc.cgi?acc=GSE20709>

| \| [**Series GSE20709**](https://www.ncbi.nlm.nih.gov/geo/query/acc.cgi?acc=GSE20709) \|  \| [Query DataSets for GSE20709](https://www.ncbi.nlm.nih.gov/gds/?term=GSE20709%5bAccession%5d) \| \| --- \| --- \| --- \| | |
| --- | --- | --- | --- | --- |
| Status | Public on Aug 27, 2010 |
| Title | Copy number data from GIST with KIT mutation |
| Organism | [Homo sapiens](https://www.ncbi.nlm.nih.gov/Taxonomy/Browser/wwwtax.cgi?mode=Info&id=9606) |
| Experiment type | Genome variation profiling by SNP array |
| Summary | In addition to KIT and PDGFRA mutations, sequential accumulation of other genetic events is involved in the development and progression of gastrointestinal stromal tumors (GISTs). Until recently, the significance of these other alterations has not been thoroughly investigated. The combination of gene expression profiling and high-resolution genomic copy number analysis offers a detailed molecular portrait of GISTs, providing an essential comprehensive knowledge necessary to guide the discovery of novel target genes involved in tumor development and progression. Fresh tissue specimens from 25 patients with GIST were collected and high-resolution genomic copy number analyses were performed using Affymetrix SNP array 6.0. |
|  |  |
| Overall design | GIST tumor samples from mutated (KIT or PDGFRA) or Wild Type patients were labeled for hybridization on Affymetrix microarrays. Copy number analysis of Affymetrix SNP6.0 arrays was performed for 25 GIST samples, then compared to gene expression data. |
|  |  |
| Contributor(s) | [Astolfi A](https://www.ncbi.nlm.nih.gov/pubmed/?term=Astolfi%20A%5bAuthor%5d), [Nannini M](https://www.ncbi.nlm.nih.gov/pubmed/?term=Nannini%20M%5bAuthor%5d), [Pantaleo MA](https://www.ncbi.nlm.nih.gov/pubmed/?term=Pantaleo%20MA%5bAuthor%5d), [Di Battista M](https://www.ncbi.nlm.nih.gov/pubmed/?term=Di%20Battista%20M%5bAuthor%5d), [Heinrich MC](https://www.ncbi.nlm.nih.gov/pubmed/?term=Heinrich%20MC%5bAuthor%5d), [Santini D](https://www.ncbi.nlm.nih.gov/pubmed/?term=Santini%20D%5bAuthor%5d), [Catena F](https://www.ncbi.nlm.nih.gov/pubmed/?term=Catena%20F%5bAuthor%5d), [Corless CL](https://www.ncbi.nlm.nih.gov/pubmed/?term=Corless%20CL%5bAuthor%5d), [Maleddu A](https://www.ncbi.nlm.nih.gov/pubmed/?term=Maleddu%20A%5bAuthor%5d), [Saponara M](https://www.ncbi.nlm.nih.gov/pubmed/?term=Saponara%20M%5bAuthor%5d), [Lolli C](https://www.ncbi.nlm.nih.gov/pubmed/?term=Lolli%20C%5bAuthor%5d), [Di Scioscio V](https://www.ncbi.nlm.nih.gov/pubmed/?term=Di%20Scioscio%20V%5bAuthor%5d), [Formica S](https://www.ncbi.nlm.nih.gov/pubmed/?term=Formica%20S%5bAuthor%5d), [Biasco G](https://www.ncbi.nlm.nih.gov/pubmed/?term=Biasco%20G%5bAuthor%5d) |
| Citation(s) | - Astolfi A, Nannini M, Pantaleo MA, Di Battista M et al. A molecular portrait of gastrointestinal stromal tumors: an integrative analysis of gene expression profiling and high-resolution genomic copy number. Lab Invest 2010 Sep;90(9):1285-94. PMID: [20548289](https://www.ncbi.nlm.nih.gov/pubmed/20548289) |
| Submission date | Mar 09, 2010 |
| Last update date | Nov 27, 2018 |
| Contact name | Maria Abbondanza Pantaleo |
| E-mail(s) | [maria.pantaleo@unibo.it](mailto:maria.pantaleo@unibo.it) |
| Organization name | Policlinico S. Orsola-Malpighi, University of Bologna |
| Department | Institute of Hematology and Medical Oncology "L.A.Seragnoli" |
| Street address | via Massarenti 9 |
| City | Bologna |
| State/province | BO |
| ZIP/Postal code | 40138 |
| Country | Italy |
|  |  |
| Platforms (1) | \| [GPL6801](https://www.ncbi.nlm.nih.gov/geo/query/acc.cgi?acc=GPL6801) \| [GenomeWideSNP_6] Affymetrix Genome-Wide Human SNP 6.0 Array \| \| --- \| --- \| |
| Samples (25)  [[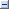](javascript:HandleVisibilityChangeL1151073059()) Less...](javascript:HandleVisibilityChangeL1151073059())  [[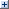](javascript:HandleVisibilityChangeL1151073059()) More...](javascript:HandleVisibilityChangeL1151073059()) | \| [GSM520391](https://www.ncbi.nlm.nih.gov/geo/query/acc.cgi?acc=GSM520391) \| GIST_02_KIT (copy number) \| \| --- \| --- \| \| [GSM520392](https://www.ncbi.nlm.nih.gov/geo/query/acc.cgi?acc=GSM520392) \| GIST_04_KIT (copy number) \| \| [GSM520393](https://www.ncbi.nlm.nih.gov/geo/query/acc.cgi?acc=GSM520393) \| GIST_05_PDGFRA (copy number) \|  \| [GSM520394](https://www.ncbi.nlm.nih.gov/geo/query/acc.cgi?acc=GSM520394) \| GIST_07_WT (copy number) \| \| --- \| --- \| \| [GSM520395](https://www.ncbi.nlm.nih.gov/geo/query/acc.cgi?acc=GSM520395) \| GIST_08_KIT (copy number) \| \| [GSM520396](https://www.ncbi.nlm.nih.gov/geo/query/acc.cgi?acc=GSM520396) \| GIST_09_KIT (copy number) \| \| [GSM520397](https://www.ncbi.nlm.nih.gov/geo/query/acc.cgi?acc=GSM520397) \| GIST_10_WT (copy number) \| \| [GSM520398](https://www.ncbi.nlm.nih.gov/geo/query/acc.cgi?acc=GSM520398) \| GIST_11_KIT (copy number) \| \| [GSM520399](https://www.ncbi.nlm.nih.gov/geo/query/acc.cgi?acc=GSM520399) \| GIST_12_PDGFRA (copy number) \| \| [GSM520400](https://www.ncbi.nlm.nih.gov/geo/query/acc.cgi?acc=GSM520400) \| GIST_13_KIT (copy number) \| \| [GSM520401](https://www.ncbi.nlm.nih.gov/geo/query/acc.cgi?acc=GSM520401) \| GIST_14_KIT (copy number) \| \| [GSM520402](https://www.ncbi.nlm.nih.gov/geo/query/acc.cgi?acc=GSM520402) \| GIST_15_PDGFRA (copy number) \| \| [GSM520403](https://www.ncbi.nlm.nih.gov/geo/query/acc.cgi?acc=GSM520403) \| GIST_16_KIT (copy number) \| \| [GSM520404](https://www.ncbi.nlm.nih.gov/geo/query/acc.cgi?acc=GSM520404) \| GIST_17_PDGFRA (copy number) \| \| [GSM520405](https://www.ncbi.nlm.nih.gov/geo/query/acc.cgi?acc=GSM520405) \| GIST_18_KIT (copy number) \| \| [GSM520406](https://www.ncbi.nlm.nih.gov/geo/query/acc.cgi?acc=GSM520406) \| GIST_19_PDGFRA (copy number) \| \| [GSM520407](https://www.ncbi.nlm.nih.gov/geo/query/acc.cgi?acc=GSM520407) \| GIST_20_KIT (copy number) \| \| [GSM520408](https://www.ncbi.nlm.nih.gov/geo/query/acc.cgi?acc=GSM520408) \| GIST_21_WT (copy number) \| \| [GSM520409](https://www.ncbi.nlm.nih.gov/geo/query/acc.cgi?acc=GSM520409) \| GIST_22_PDGFRA (copy number) \| \| [GSM520410](https://www.ncbi.nlm.nih.gov/geo/query/acc.cgi?acc=GSM520410) \| GIST_23_KIT (copy number) \| \| [GSM520411](https://www.ncbi.nlm.nih.gov/geo/query/acc.cgi?acc=GSM520411) \| GIST_24_WT (copy number) \| \| [GSM520412](https://www.ncbi.nlm.nih.gov/geo/query/acc.cgi?acc=GSM520412) \| GIST_25_KIT (copy number) \| \| [GSM520413](https://www.ncbi.nlm.nih.gov/geo/query/acc.cgi?acc=GSM520413) \| GIST_26_PDGFRA (copy number) \| \| [GSM520414](https://www.ncbi.nlm.nih.gov/geo/query/acc.cgi?acc=GSM520414) \| GIST_27_KIT (copy number) \| \| [GSM520415](https://www.ncbi.nlm.nih.gov/geo/query/acc.cgi?acc=GSM520415) \| GIST_28_KIT (copy number) \| |
| This SubSeries is part of SuperSeries: | |
| \| [GSE20710](https://www.ncbi.nlm.nih.gov/geo/query/acc.cgi?acc=GSE20710) \| Integrative analysis of gene expression profiling and genomic copy number in Gastrointestinal Stromal Tumors \| \| --- \| --- \| | |
| **Relations** | |
| BioProject | [PRJNA129575](https://www.ncbi.nlm.nih.gov/bioproject/PRJNA129575) |

| **Download family** | **Format** |
| --- | --- |
| [SOFT formatted family file(s)](https://ftp.ncbi.nlm.nih.gov/geo/series/GSE20nnn/GSE20709/soft/) | SOFT[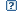](javascript:RPopUpWindow_Set(geoaxema_famsoft,260,120,'','','#E1EAE6','','#538AA9','MessageBox2');) |
| [MINiML formatted family file(s)](https://ftp.ncbi.nlm.nih.gov/geo/series/GSE20nnn/GSE20709/miniml/) | MINiML[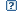](javascript:RPopUpWindow_Set(geoaxema_famminiml,260,120,'','','#E1EAE6','','#538AA9','MessageBox2');) |
| [Series Matrix File(s)](https://ftp.ncbi.nlm.nih.gov/geo/series/GSE20nnn/GSE20709/matrix/) | TXT[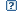](javascript:RPopUpWindow_Set(geoaxema_fammatrix,260,210,'','','#E1EAE6','','#538AA9','MessageBox2');) |

| **Supplementary file** | **Size** | **Download** | **File type/resource** |
| --- | --- | --- | --- |
| GSE20709_RAW.tar | 708.0 Mb | [(http)](https://www.ncbi.nlm.nih.gov/geo/download/?acc=GSE20709&format=file)(custom) | TAR (of CEL) |
